# Chronic high-fat diet induces overeating and impairs synaptic transmission in feeding-related brain regions

**DOI:** 10.3389/fnmol.2022.1019446

**Published:** 2022-09-26

**Authors:** Xu Wang, Haohong Li

**Affiliations:** ^1^Britton Chance Center for Biomedical Photonics, Wuhan National Laboratory for Optoelectronics, Huazhong University of Science and Technology, Wuhan, China; ^2^Ministry of Education (MoE) Key Laboratory for Biomedical Photonics, Collaborative Innovation Center for Biomedical Engineering, School of Engineering Sciences, Huazhong University of Science and Technology, Wuhan, China; ^3^Affiliated Mental Health Centre and Hangzhou Seventh People’s Hospital, Zhejiang University School of Medicine, Hangzhou, China; ^4^The Ministry of Education (MoE) Frontier Research Center of Brain and Brain-machine Integration, Zhejiang University School of Brain Science and Brain Medicine, Hangzhou, China

**Keywords:** high-fat diet, third ventricles, synaptic transmission, arcuate nucleus, ventromedial hypothalamus, lateral hypothalamus, paraventricular nucleus of the hypothalamus, nucleus accumbens

## Abstract

Obesity is linked to overeating, which can exacerbate unhealthy weight gain. However, the mechanisms for mediating such linkages are elusive. In the current study, we hypothesized that synaptic remodeling occurs in feeding-related brain regions of obese mice. To investigate this, we established a high-fat diet (HFD)-induced obese mouse model and observed that these mice consumed excessive calories. The effect of chronic HFD feeding on lipid droplet accumulation in different brain structures was also investigated. We found that lipid droplets accumulated on the ependyma of the third ventricle (3V), which is surrounded by key areas of the hypothalamus that are involved in feeding. Then, the spontaneous synaptic activity of miniature excitatory postsynaptic current (mEPSC) and miniature inhibitory postsynaptic current (mIPSC) was recorded in these hypothalamic areas. HFD induced a decreased amplitude of mEPSC in the arcuate nucleus (ARC) and the ventromedial hypothalamus (VMH), meanwhile, increased the frequency in the VMH. In addition, HFD reduced the frequency of mIPSC in the lateral hypothalamus (LH) and increased the amplitude of mIPSC in the paraventricular nucleus of the hypothalamus (PVH). Subsequently, we also measured the synaptic activity of nucleus accumbens (NAc) neurons, which play a vital role in the hedonic aspect of eating, and discovered that HFD diminished the frequency of both mEPSC and mIPSC in the NAc. These findings suggest that chronic HFD feeding leads to lipid accumulation and synaptic dysfunction in specific brain regions, which are associated with energy homeostasis and reward regulation, and these impairments may lead to the overeating of obesity.

## Introduction

Obesity, often associated with chronic disease and disability, has reached global epidemic levels (Danaei et al., 2011). Overconsumption of energy-dense foods is often cited as a major contributing factor in unhealthy weight gain, but hyperphagia is not only a cause of weight gain but also a common symptom of obesity (Morton et al., 2006). The central nervous system (CNS) plays a key role in maintaining energy homeostasis by integrating external and internal stimuli to enact metabolic and behavioral responses. We hypothesized that the dysfunction of energy homeostatic regulation may have occurred in the CNS after long-term overeating. However, how hyperphagic obesity induces neural remodeling remains largely obscure.

The hypothalamus is a critical brain structure for this homeostatic regulation, integrating hormonal, behavioral, and autonomic responses to coordinate intake and consumption (Gao and Horvath, 2007; Coll and Yeo, 2013). The arcuate nucleus (ARC) is a necessary sensory region that receives neuronal and humoral signals. Neurons in the ARC express receptors for circulating hormones, including leptin, ghrelin and insulin (Rossi and Stuber, 2018). Agouti-related peptide (AgRP) and proopiomelanocortin (POMC) neurons are intermingled but non-overlapped neurons in the Arc, and have been shown to be involved in energy homeostatic regulation. AgRP neurons output an orexigenic signal, and POMC neurons are considered functionally opposite, the balance between these two groups of neurons is essential for maintaining energy homeostasis in the body (Betley et al., 2013; Andermann and Lowell, 2017). Moreover, AgRP neurons send projections to multiple regions of the hypothalamus, including the paraventricular nucleus of the hypothalamus (PVH) and lateral hypothalamus (LH), activating either ARC > PVH or ARC > LH projection is sufficient to induce feeding (Betley et al., 2013). Conversely, inhibited mediation of AgRP neurons to PVH neurons prevents feeding behaviors, and PVH lesions lead to hyperphagic obesity (Atasoy et al., 2012). In addition, as a major output site of the hypothalamus to the hindbrain, the PVH is essential for leptin to control energy balance (Sawchenko and Swanson, 1982; Wu et al., 2009). PVH neurons are innervated by LH GABAergic neurons, and optogenetic stimulation of this circuit can increase feeding behavior (Wu et al., 2015). Nevertheless, activating glutamatergic neurons in the LH has the opposite effect (Jennings et al., 2013). The ventromedial hypothalamus (VMH) is another hypothalamic region that expresses leptin receptors. Research has shown that the deletion of steroidogenic factor 1 (SF1)-specific leptin receptors predisposes mice to obesity and metabolic syndrome (Dhillon et al., 2006). VMH neurons receive projections from POMC neurons and further project back to the ARC. Specifically, VMH glutamatergic neurons transduce excitatory input to both POMC and AgRP neurons, and loss of these inputs induces an increase in feeding behaviors (Tong et al., 2007). Therefore, hypothalamic connections are significantly plasticity regulated by peripheral hormones and nutrients.

In addition to energy homeostatic needs, the drive to eat is also governed by the rewarding properties of food. A previous human study found that hedonic feeding is significantly higher in obese individuals than in lean individuals (Rabiei et al., 2019). The reward system involves the release of dopamine that originates from the ventral tegmental area (VTA) and projects to the nucleus accumbens (NAc), striatum and other brain regions (Kelley and Berridge, 2002). It has been reported that orexin neurons from the LH activate dopaminergic neurons in the VTA, which in turn stimulates the VTA > NAc pathway and promotes palatable food consumption (Baik, 2013). The adverse effects of obesity on these feeding-related brain regions are unclear. We hypothesized that obesity may disrupt the balance between orexigenic and anorexigenic neurons, leading to excessive energy intake. Synaptic plasticity is an ability of neuronal activity that needs to adapt in an experience-dependent manner (Feldman, 2009). We further hypothesized that obesity remodels synaptic plasticity in feeding-related brain regions, which may lead to hyperphagia.

As such, we found that the high-fat diet (HFD)-induced obese mice ingested excess calories and exhibited reduced physical activity. In addition, their glucose and insulin metabolism were abnormal. To understand how obesity affects the CNS, we first quantified the distribution of lipid droplets in several brain structures and found large amounts in the ependyma of the third ventricle (3V). Moreover, synaptic activity in the hypothalamus and NAc were tested in HFD-fed mice, and impaired excitatory and inhibitory synaptic transmission was observed in these areas. These results highlight the dysfunction of synaptic transmission in feeding-related brain regions of obesity, which may be in part linked with lipid accumulation in the 3V, and suggest potential neural mechanisms of obesity-related hyperphagia.

## Materials and methods

### Animals

Seven-week-old male C57BL/6J mice were allocated into two groups with *ad libitum* (AL) access to either normal diet (ND, 1,032, Beijing HFK Bioscience, 20% protein, 4% fat, 68% carbohydrates) or HFD (MD12033, Medicine, 20% protein, 60% fat, 20% carbohydrates) for 12 weeks. Then, locomotor activity, body weight, food intake, and synaptic transmission were all measured after 12 weeks of feeding treatment. Mice were kept in the Wuhan National Laboratory with regular temperature (22 ± 2°C), humidity (40–60%), and circadian cycle [12:12 light:dark (LD) cycle, with light on at zeitgeber time 0 (ZT 0, 7:00 a.m.)]. Water was provided AL. All experiments were performed in accordance with the guidelines of the Institute of Neuroscience, Chinese Academy of Sciences and University. All procedures involving animals were approved by the Hubei Provincial Animal Care and Use Committee and were in accordance with the experimental guidelines of the Animal Experimentation Ethics Committee of Huazhong University of Science and Technology, China.

### Food intake

Initially, mice were single housed for at least 7 days with a 12:12 LD cycle. Food and water were available AL. Then, food intake was calculated as the difference in weight of food every 12 h, starting at lights on (ZT0).

### Glucose tolerance tests and insulin tolerance tests

Mice were fasted for 16 or 4 h for GTTs or ITTs, respectively. Blood glucose was measured using a Glucometer (Accu-Chek Active) by tail bleeds. Mice were intraperitoneally injected with glucose (1 g/kg of body weight in saline) or insulin (0.75 U/kg body weight in saline). Blood glucose was continuously monitored several times after injection as indicated (Grippo et al., 2020).

### Open-field activity

To evaluate locomotor activity, mice were placed in an acrylic arena (40 × 40 × 45 cm^3^) for 15 min. The tracks were recorded with a Logitech C1000e camera, and idTracker software^1^ was used to track mice throughout testing.

### Oil red O

Coronal 30 μm slices of ND and HFD mouse brains were washed in PBS and 60% isopropanol, and then the slices were incubated in 0.3% oil red O in isopropanol (Sigma-Aldrich) for 30 min. Images were taken on Tinyscope Exos001 (Convergence.tech) and processed using ImageJ software.^2^

### Patch clamp recording

Mice were deeply anesthetized with isoflurane, and their brains were rapidly dissected from the skulls. Then, the brains were placed in cutting buffer containing the following (in mM): 2.5 KCl, 0.5 CaCl_2_, 7.2 MgCl_2_, 25 NaHCO_3_, 1.1 NaH_2_PO_4_, 25 D-glucose, 11 sodium ascorbate, 3 sodium pyruvate, and 97 choline chloride. Coronal slices (300 μm) were cut in 95% O_2_/5% CO_2_-oxygenated cutting buffer with a vibratome (Leica VT1000S, Germany). Slices were incubated at 32°C in a submerged chamber containing artificial cerebrospinal fluid (ACSF) equilibrated with 95% O_2_/5% CO_2_ for at least 30 min, followed by recovery for 30 min at room temperature. The ACSF contained the following (in mM): 118 NaCl, 2.5 KCl, 2 CaCl_2_, 2 MgCl_2_, 26 NaHCO_3_, 0.9 NaH_2_PO_4_, and 11 D-glucose. The slices bubbled with 95% O_2_/5% CO_2_ were transferred to a recording chamber. Patch-clamp electrodes were pulled from borosilicate glass (1.5 mm diameter, VitalSense, B15024N) on a three-stage puller (Sutter P-1000). The pipettes had a resistance of 2–6 MΩ. Recordings were collected using Axon Multiclamp 700B amplifiers, Digidata 1440A (Molecular Devices), and analyzed using MiniAnalysis software (Synaptosoft). During recordings, series resistance was continuously monitored. Recordings with series resistance of > 25 MΩ were excluded from analysis. We did not compensate for the series resistance, but cells in which Rs changed by > 15% were discarded. The pipette solution contained the following (in mM): 140 K-gluconate, 0.1 CaCl_2_, 2 MgCl_2_, 1 EGTA, 2 ATP K_2_, 0.1 GTP Na_3_, and 10 HEPES (pH = 7.25, osmolality 305 mOsm). The miniature excitatory postsynaptic current (mEPSC) and miniature inhibitory postsynaptic current (mIPSC) were recorded for 10 min at a holding potential of −70 or 0 mV, respectively. All experiments were performed in the presence of tetrodotoxin (TTX, 1 μM). And the recording sites of different brain regions are shown in Supplementary Figure S1.

### Statistics analysis

Statistical tests included the Mann-Whitney test, two-tailed unpaired *t*-tests, and two-way ANOVA followed by Bonferroni *post hoc* analysis. Analyses were conducted using GraphPad Prism 8 (GraphPad Software) statistical software for Windows or MATLAB R2014b (MathWorks). Data are expressed as the mean ± SEM. Statistical significance was set at *p < 0.05; ^**^p < 0.01; ^***^p < 0.001.

## Results

### Lipid droplets accumulate in the ependyma of the third ventricle in chronic high-fat diet-exposed mice

**FIGURE 1:**
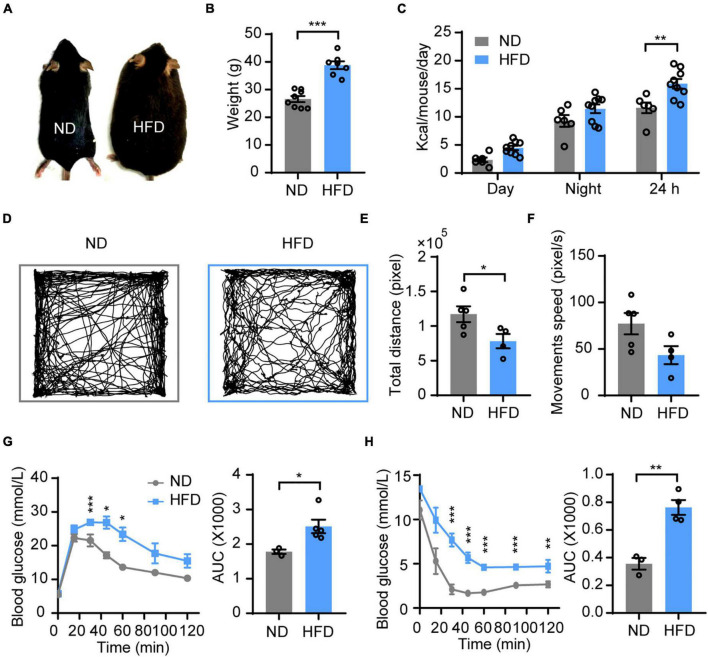
Chronic HFD leads to weight gain, overeating, physical inactivity, and metabolic disorders. **(A)** Photo of representative ND and HFD mice. **(B)** Average body weight of ND and HFD mice (*n* = 7–8, two-tailed unpaired *t-*test). **(C)** Food ingested during the day (left), night (middle), and 24 h (right) for ND and HFD mice (*n* = 6–9, two-way ANOVA, Bonferroni *post hoc* analysis). **(D)** Representative track plots of open field activity for ND and HFD mice. **(E,F)** The total traveled distance **(E)** and movement speed **(F)** of ND and HFD mice. **(G)** Blood glucose levels during the glucose tolerance test (left, GTT) and area under the curve (right, AUC) in ND and HFD mice (*n* = 3–6, two-tailed unpaired *t-*test). **(H)** Blood glucose levels during the insulin tolerance test (left, ITT) and area under the curve (right, AUC) in ND and HFD mice (*n* = 3–4, two-tailed unpaired *t-*test). Data are means ± SEMs. Dots represent individual experimental animals. *p < 0.05; **p < 0.01; ***p < 0.001 as indicated.

To investigate the neural mechanisms underlying the overeating of obesity, C57BL6/J male mice (7 weeks) were AL fed either a ND or a HFD for 12 weeks. Compared to the ND group, the HFD group showed significant weight gain (Figures 1A,B). This symptom was induced by a positive energy balance, which was reflected in an increase in daily energy intake (Figure 1C) and a decrease in physical activity (Figures 1D–F). Moreover, chronic HFD consumption induced two hallmarks of obesity-related metabolic diseases, including elevated glucose tolerance and insulin resistance (Figures 1G,H).

**FIGURE 2:**
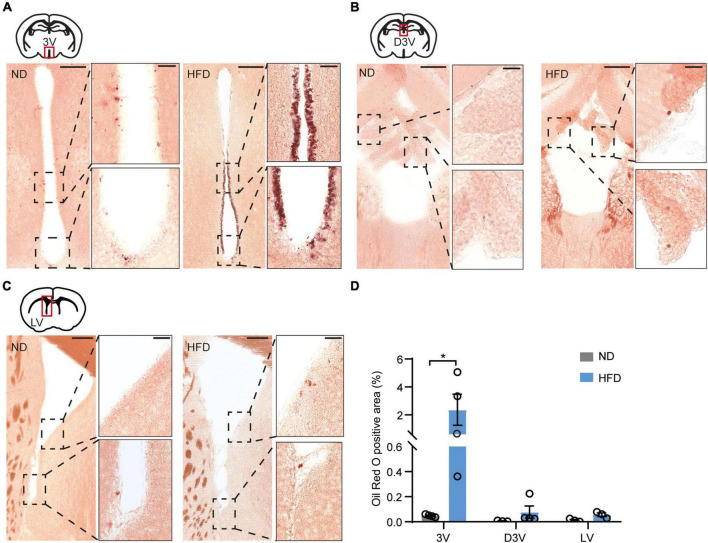
Chronic HFD induces the accumulation of lipid droplets in the ependyma of the third ventricles. **(A)** Representative images showing lipid droplets accumulated in the ependyma of 3V, high-magnification micrographs are on the right side. **(B)** Representative images showing few lipid droplets accumulated in the choroid plexus of the D3V, high-magnification micrographs are on the right side. **(C)** Representative images showing few lipid droplets accumulated in the LV ependyma and surrounding areas; high-magnification micrographs are on the right side. The bar represents 150/30 μm (for magn). **(D)** Quantification of oil red O staining, which was performed using ImageJ software. Mann-Whitney test, *n* = 3–4 mice. Data are means ± SEMs. Dots represent individual experimental animals. *p < 0.05 as indicated.

Using these HFD-induced obese mice, we investigated obesity-induced lipotoxicity in the brain, referring to an ectopic accumulation of lipid droplets in the non-adipose tissues, which is considered to contribute to organ injuries in the context of metabolic diseases (Saponaro et al., 2015). Oil red O staining was used to delineate intracellular neutral lipids in distinct brain structures. The staining showed accumulation of lipid droplets in the ependyma of the 3V of HFD mice (Figure 2A). In addition, few lipid droplets were observed in the choroid plexus along the dorsal third ventricles (D3V) and the ependyma of the lateral ventricles (LV) (Figures 2B,C). According to the statistics, lipid droplets accumulated significantly more in the ependyma of the 3V than in the D3V or the LV (Figure 2D). 3V is adjacent to many regions of the hypothalamus involved in regulating feeding behaviors (Morton et al., 2006). Lipid accumulation in the 3V may form a brain-cerebrospinal fluid (CSF) barrier that prevents energy metabolic signals from passing through the CSF to deeper hypothalamic regions. These findings strongly imply that obesity-induced lipid accumulation may damage hypothalamic function.

### High-fat diet impairs synaptic transmission in various hypothalamic regions

Energy homeostasis is regulated by sensing and integrating nutritional and hormonal signals from the periphery, a process involving a complex CNS network to coordinate subsequent feeding behavior, energy expenditure, and glucose homeostasis. The hypothalamus is a well-established brain site involved in regulating the energy balance exerted by the periphery (Blouet and Schwartz, 2010). We verified that lipid droplets accumulated in the ventricles near the hypothalamus (3V). Electrophysiology was then performed *in vitro* to examine how HFD feeding affects inputs to the hypothalamic regions, which may lead to overeating.

**FIGURE 3:**
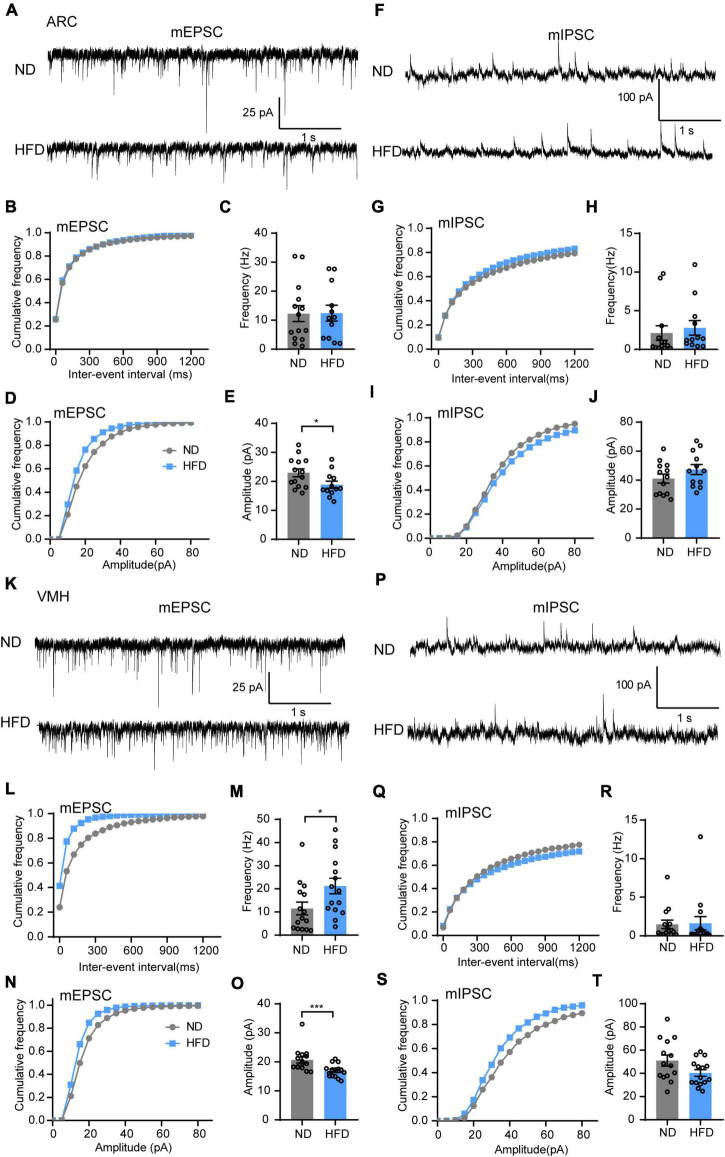
Chronic HFD consumption impairs excitatory synaptic transmission of the VMH and ARC. **(A)** Representative mEPSC traces recorded from ARC neurons in ND and HFD mice. **(B,C)** Cumulative fraction curves of inter-event intervals **(B)** and mean frequency **(C)** of mEPSCs (*n* = 3 mice, 12–14 cells, two-tailed unpaired *t-*test). **(D,E)** Cumulative fraction curves of amplitudes **(D)** and mean amplitude **(E)** of mEPSCs (*n* = 3 mice, 12–14 cells, two-tailed unpaired *t-*test). **(F)** Representative mIPSC traces recorded from ARC neurons in ND and HFD mice. **(G,H)** Cumulative fraction curves of inter-event intervals **(G)** and mean frequency **(H)** of mIPSCs (*n* = 3 mice, 12–13 cells, Mann-Whitney test). **(I,J)** Cumulative fraction curves of amplitudes **(I)** and mean amplitude **(J)** of mIPSCs (*n* = 3 mice, 12–13 cells, two-tailed unpaired *t-*test). **(K)** Representative mEPSC traces recorded from VMH neurons for ND and HFD mice. **(L,M)** Cumulative fraction curves of inter-event intervals **(L)** and mean frequency **(M)** of mEPSCs (*n* = 3 mice, 15 cells, Mann-Whitney test). **(N,O)** Cumulative fraction curves of amplitudes **(N)** and mean amplitude **(O)** of mEPSCs (*n* = 3 mice, 15 cells, Mann-Whitney test). **(P)** Representative mIPSC traces recorded from VMH neurons for ND and HFD mice. **(Q,R)** Cumulative fraction curves of inter-event intervals **(Q)** and mean frequency **(R)** of mIPSCs (*n* = 3 mice, 14–15 cells, Mann-Whitney test). **(S,T)** Cumulative fraction curves of amplitudes **(S)** and mean amplitude **(T)** of mIPSCs (*n* = 3 mice, 14–15 cells, two-tailed unpaired *t-*test). Data are means ± SEMs. Dots represent individual experimental cells. *p < 0.05; ***p < 0.001 as indicated.

First, we hypothesized that lipid droplet accumulation affects hypothalamic neurons that express receptors for circulating hormones. The synaptic transmission of the ARC and VMH, which are major regions of leptin and insulin receptor expression, was tested (Paranjape et al., 2010; Harwood, 2012; Kleinridders et al., 2014; van Swieten et al., 2014). Using *in vitro* patch clamp recordings, we measured mEPSC and mIPSC in the ARC and VMH neurons in the presence of TTX (1 μM). Interestingly, HFD feeding significantly reduced the mEPSC amplitude of ARC neurons, but there was no difference in mEPSC frequency between ND and HFD mice (Figures 3A–E). Similar suppressive effects were also present in the amplitude of mEPSC for the VMH after chronic HFD exposure. Meanwhile, HFD increased the frequency of mEPSC in VMH neurons (Figures 3K–O). However, no differences were observed in the amplitude and frequency of mIPSC in the ARC and VMH neurons (Figures 3F–J,P–T). Changes in the amplitude of spontaneous events are interpreted as reflecting postsynaptic mechanisms. The amplitude analysis revealed that chronic HFD exposure disrupted the excitatory synaptic transmission likely through decreasing the presence of postsynaptic receptors or vesicle transmitter contents in the ARC and VMH. This may be affected by the accumulation of lipid droplets in the 3V, which possibly induced insulin and leptin resistance in these hypothalamic regions. Nevertheless, HFD mice also had an increased frequency of mEPSC in the VMH, which is interpreted as an enhanced presynaptic release probability. This may be due to dysfunctions of inter- or extrahypothalamic connections, which could lead to the dysregulation of autonomic sympathetic nervous outflow in obese patients (Cristino et al., 2013).

**FIGURE 4:**
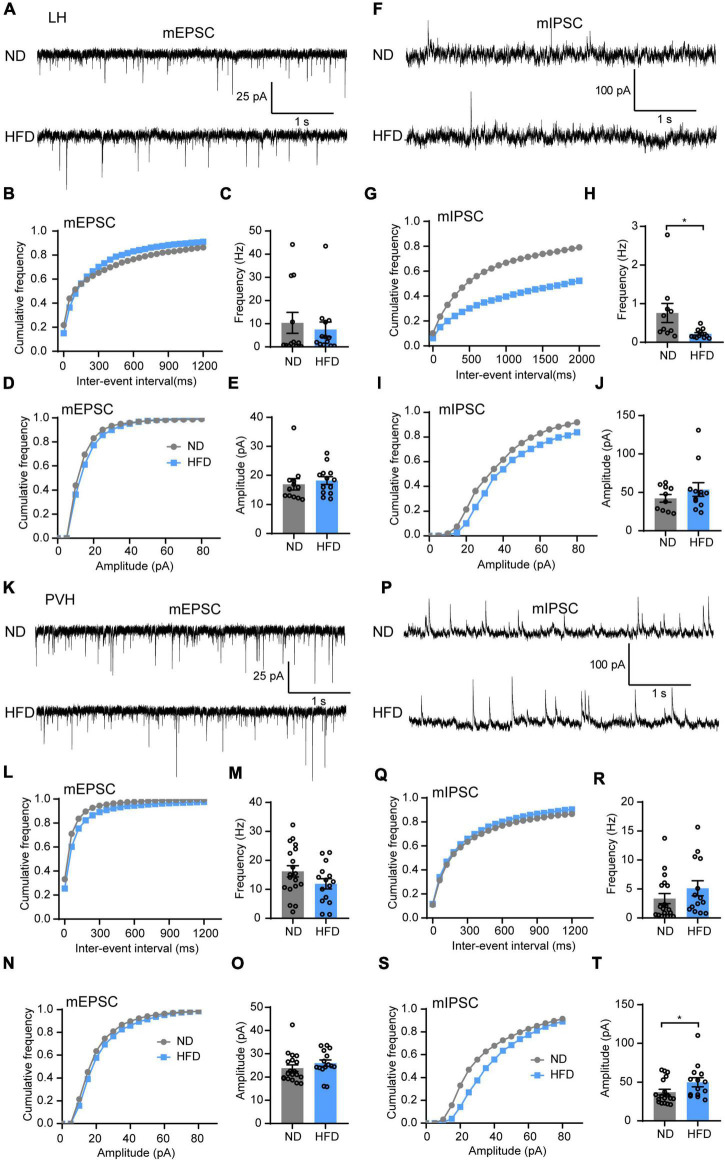
Chronic HFD consumption impairs inhibitory synaptic transmission of the LH and PVH. **(A)** Representative mEPSC traces recorded from LH neurons for ND and HFD mice. **(B,C)** Cumulative fraction curves of inter-event intervals **(B)** and mean frequency **(C)** of mEPSCs (*n* = 3–4 mice, 12–13 cells, Mann-Whitney test). **(D,E)** Cumulative fraction curves of amplitudes **(D)** and mean amplitude **(E)** of mEPSCs (*n* = 3–4 mice, 12–13 cells, Mann-Whitney test). **(F)** Representative mIPSC traces recorded from LH neurons for ND and HFD mice. **(G,H)** Cumulative fraction curves of inter-event intervals **(G)** and mean frequency **(H)** of mIPSCs (*n* = 3–4 mice, 10–12 cells, Mann-Whitney test). **(I,J)** Cumulative fraction curves of amplitudes **(I)** and mean amplitude **(J)** of mIPSCs (*n* = 3–4 mice, 10–12 cells, Mann-Whitney test). **(K)** Representative mEPSC traces recorded from PVH neurons for ND and HFD mice. **(L,M)** Cumulative fraction curves of inter-event intervals **(L)** and mean frequency **(M)** of mEPSCs (*n* = 3–4 mice, 15–19 cells, two-tailed unpaired *t-*test). **(N,O)** Cumulative fraction curves of amplitudes **(N)** and mean amplitude **(O)** of mEPSCs (*n* = 3–4 mice, 15–19 cells, Mann-Whitney test). **(P)** Representative mIPSC traces recorded from LH neurons for ND and HFD mice. **(Q,R)** Cumulative fraction curves of inter-event intervals **(Q)** and mean frequency **(R)** of mIPSCs (*n* = 3–4 mice, 14–18 cells, Mann-Whitney test). **(S,T)** Cumulative fraction curves of amplitudes **(S)** and mean amplitude **(T)** of mIPSCs (*n* = 3–4 mice, 14–18 cells, Mann-Whitney test). Data are means ± SEMs. Dots represent individual experimental cells. *p < 0.05 as indicated.

Except for the ARC and VMH, there are other regions of the hypothalamus that sense changes in nutritional status and dynamically adjust food intake to maintain energy homeostasis. The LH and PVH are involved in mediating feeding behaviors and energy expenditure (Margules and Olds, 1962; Sutton et al., 2016; Rossi and Stuber, 2018). Hence, we further tested how chronic HFD feeding affects synaptic transmission of LH and PVH neurons. The results showed that HFD did not alter the amplitude and frequency of mEPSC (Figures 4A–E) but decreased the frequency of mIPSC in the LH (Figures 4F–H). Nevertheless, HFD did not affect the mIPSC amplitude of the LH (Figures 4I,J). This finding indicates a reduced presynaptic release probability of inhibitory synapses in the LH. Furthermore, there was no remarkable difference in the excitatory synaptic transmission of PVH neurons between ND and HFD mice (Figures 4K–O), but a decreased amplitude of mIPSC in the PVH was observed in the HFD group (Figures 4P–T), which is considered a postsynaptic mechanism. Together, these results led us to determine that a chronic HFD induced synaptic transmission disorder in multiple hypothalamic areas associated with energy homeostatic regulation.

### High-fat diet impairs synaptic transmission in the nucleus accumbens

**FIGURE 5:**
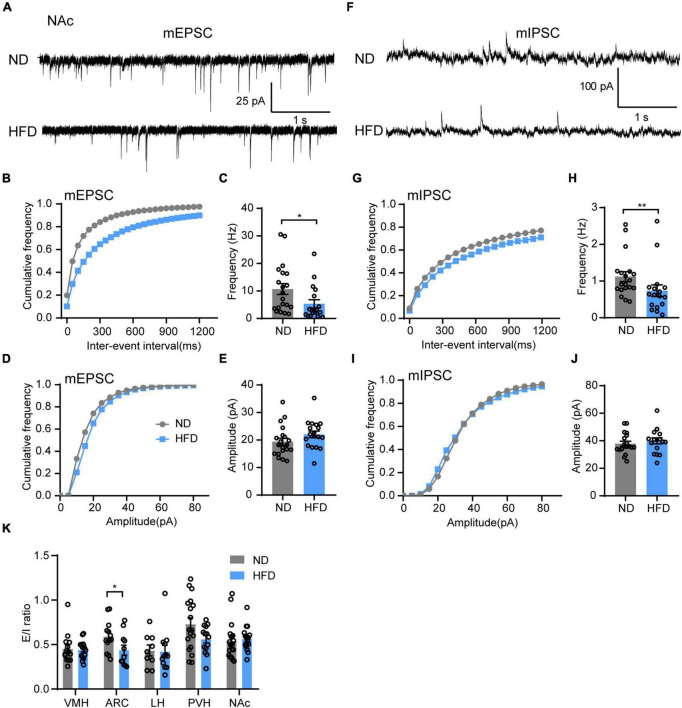
Chronic HFD consumption impairs synaptic transmission in the NAc. **(A)** Representative mEPSC traces recorded from NAc neurons for ND and HFD mice. **(B,C)** Cumulative fraction curves of inter-event intervals **(B)** and mean frequency **(C)** of mEPSCs (*n* = 3 mice, 18–20 cells, Mann-Whitney test). **(D,E)** Cumulative fraction curves of amplitudes **(D)** and mean amplitude **(E)** of mEPSCs (*n* = 3 mice, 18–20 cells, two-tailed unpaired *t-*test). **(F)** Representative mIPSC traces recorded from NAc neurons for ND and HFD mice. **(G,H)** Cumulative fraction curves of inter-event intervals **(G)** and mean frequency **(H)** of mIPSCs (*n* = 3 mice, 17–19 cells, Mann-Whitney test). **(I,J)** Cumulative fraction curves of amplitudes **(I)** and mean amplitude **(J)** of mIPSCs (*n* = 3 mice, 17–19 cells, two-tailed unpaired *t-*test). **(K)** Ratio between mEPSC and mIPSC amplitudes in the same neurons from different brain regions in ND and HFD mice (*n* = 3–4 mice, 9–19 cells, Mann-Whitney test). Data are means ± SEMs. Dots represent individual experimental cells. *p < 0.05; **p < 0.01 as indicated.

Motivational circuitry interacts with hypothalamic energy homeostatic circuitry to coordinate feeding behavior (Baik, 2013; Liu et al., 2020). In addition to energy homeostatic dysregulation, hedonic eating is also a major contributor to overeating for obesity, which is considered to damage the reward system. Brain hedonic circuitry focuses on stimulating the mesolimbic dopamine pathway to enhance signaling to the NAc (Gendelis et al., 2021). To test the effects of HFD feeding on the food reward system, we next tested synaptic transmission in the NAc. Indeed, chronic HFD consumption reduced the frequency of both mEPSC (Figures 5B,C) and mIPSC (Figures 5F–H) in the NAc. However, there was no significant difference in the amplitude of mEPSC (Figures 5D,E) or mIPSC (Figures 5I,J) between the ND and HFD groups. This indicates that HFD-induced obesity disrupts synaptic transmission of the NAc likely through impairing presynaptic release probability, which may be associated with hedonic eating in obese mice.

Finally, we demonstrated whether chronic HFD-induced obesity alters the balance of excitatory and inhibitory inputs in different brain regions. The mEPSC and mIPSC amplitude ratio of the same neurons was calculated to identify the relationship between synaptic excitation and inhibition (E/I ratio) of different regions for ND and HFD mice. The HFD group had a lower E/I ratio in the ARC but no significant effect on other brain regions (Figure 5K).

**FIGURE 6:**
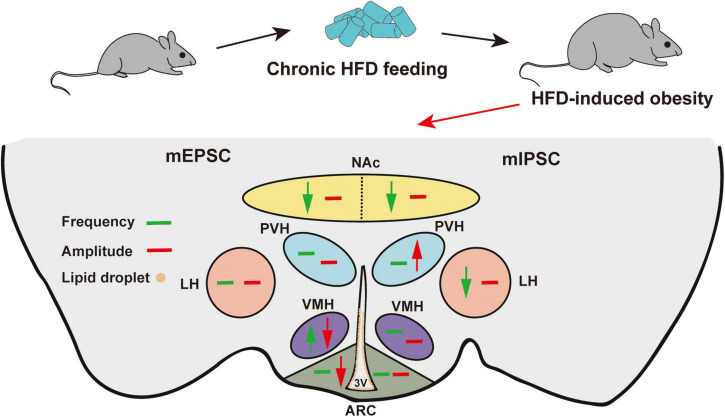
Schematic model of HFD-induced changes in synaptic transmission in different brain regions. Green arrows indicate significant changes in frequency. Red arrows indicate significant changes in amplitude. Brown circles indicate lipid droplet accumulation. The mEPSC changes are on the left, and the mIPSC changes are on the right. Chronic HFD consumption induced lipid droplet accumulation in the 3V and impaired the synaptic transmission of feeding-related brain regions.

We summarized the effects of chronic HFD-induced obesity on excitatory and inhibitory synaptic transmission in brain regions, which integrate external and internal information to maintain energy balance (Figure 6 and Table 1). These results strongly imply that the damage to the brain regions associated with feeding in the CNS may be the cause of obesity-related overeating.

**TABLE 1:** Summary of the frequency, amplitude and E/I ratio of mEPSCs and mIPSCs in ND, and HFD mice.

			VMH	ARC	LH	PVH	NAc
mEPSC	Frequency	ND	11.49 ± 2.73	12.26 ± 2.76	10.37 ± 4.52	16.22 ± 1.95	10.68 ± 1.97
		HFD	21.19 ± 3.37*	12.42 ± 2.73	7.53 ± 3.22	11.92 ± 1.87	5.35 ± 1.46*
	Amplitude	ND	20.64 ± 1.04	22.95 ± 1.38	16.93 ± 1.93	23.78 ± 1.41	19.39 ± 1.23
		HFD	16.90 ± 0.58**	18.84 ± 1.23*	18.19 ± 1.34	25.92 ± 1.41	22.04 ± 1.21
mIPSC	Frequency	ND	1.49 ± 0.55	2.12 ± 0.94	0.76 ± 0.25	3.35 ± 0.88	1.12 ± 0.13
		HFD	1.62 ± 0.85	2.78 ± 0.95	0.22 ± 0.03*	5.13 ± 1.29	0.74 ± 0.16**
	Amplitude	ND	51.00 ± 4.78	41.06 ± 3.09	42.31 ± 5.05	37.12 ± 3.73	37.91 ± 1.73
		HFD	40.35 ± 2.87	47.31 ± 3.47	53.65 ± 8.73	49.93 ± 5.87*	39.96 ± 2.17
E/I ratio		ND	0.45 ± 0.05	0.59 ± 0.05	0.73 ± 0.07	0.43 ± 0.06	0.54 ± 0.05
		HFD	0.44 ± 0.02	0.43 ± 0.05*	0.56 ± 0.04	0.42 ± 0.07	0.56 ± 0.03

Data are means ± SEMs. *p < 0.05; **p < 0.01 as indicated.

## Discussion

Most previous research on the metabolic diseases of obesity has focused on peripheral tissues, such as pancreatic β cells, liver, and heart, with little known about how synaptic plasticity in the CNS is associated with obesity. Synaptic plasticity is altered in response to experience, which promotes animal survival under different conditions. Additionally, synaptic plasticity is essential for regulating energy intake and expenditure to maintain energy balance in most adult mammals (Horvath, 2006). Overeating and hedonic eating are two critical contributors to obesity, and obesity-related hormonal dysfunction often leads to a strong physiological drive to eat more. In addition to notable hormone disorders, overeating may have significant neurobiological underpinnings, which are not simply linked with damage to individual brain regions but an imbalance of feeding-related neural networks. We hypothesized that these circuits fail to adapt in some changing environments, calorically dense, and HFD may lead to hypothalamic circuit dysfunction, mismatching energy availability to needs, thereby impairing the reward system and leading to obesity and type 2 diabetes. In the current study, we found that HFD-induced obesity impairs synaptic transmission in multiple brain regions associated with energy homeostasis and reward regulation, and these results further imply the CNS mechanisms of overeating induced by obesity.

A model of energy homeostasis proposes that body adiposity is regulated by an endocrine “adiposity” negative feedback loop, including hormones of leptin and insulin (Seeley et al., 1996). Leptin receptors are highly expressed in the hypothalamic brains, especially in the ARC and VMH, while their expression in the PVN and LH is lower (Harwood, 2012; van Swieten et al., 2014). Leptin transmits the satiety signal originating from adipose tissue, stimulates the hypothalamic leptin receptors and further inhibits feeding behavior (Wilson and Enriori, 2015; Zeng et al., 2022). Likewise, insulin receptors are also expressed in ARC and VMH neurons (Paranjape et al., 2010; Kleinridders et al., 2014). Insulin, a hormone secreted by the pancreas, activates hypothalamic downstream signaling pathways to restrict food intake. HFD-induced obesity is often associated with insulin and leptin resistance (Paranjape et al., 2011; Zhang et al., 2014). Obesity induces lipid accumulation in multiple organs and has been studied in peripheral tissues (Kan et al., 2020). Until now, the effects of obesity-related lipid accumulation in the CNS have not been fully understood. In the present research, we found that lipid droplets accumulated significantly more in the ependyma of the 3V than in the D3V or the LV in HFD-induced obese mice (Figure 2). A previous study revealed that senescent glial cells accumulate lipids in the subventricular zone, including areas around the LV, the 3V, the fourth ventricles (4V), and the periaqueductal gray (PAG) matter (Ogrodnik et al., 2019). However, our testing of lipid accumulation focused on the ependyma, which may affect the signal transmissions from the CSF. The effect of HFD on molecular transport in the hypothalamus is poorly characterized. The lipotoxicity in the hypothalamus is inextricably linked to inflammation and reactive gliosis. These hypothalamic disorders contribute to leptin and insulin resistance, which are associated with the development of obesity (De Souza et al., 2005; Zhang and Kaufman, 2008; Valdearcos et al., 2014). On the other hand, tanycytes, the radial glia-like cells in the ependyma of the 3V, act as gatekeepers to circulating signals and form a blood-CSF barrier. Tanycytes transport blood-borne metabolic signals such as leptin and ghrelin in CSF and integrate signals to regulate appetite and energy balance (Bolborea and Dale, 2013; Folick et al., 2021). Increasing evidence points out that lesions of tanycytes hamper its anti-obesity effects on food intake, body weight and fat mass (Duquenne et al., 2021; Imbernon et al., 2022). Thus, we suspect that HFD-induced lipid accumulation in the ependyma of the 3V may result in tanycytes dysfunction, brakes hypothalamic neurons access to blood-borne metabolic signals, and contributes to leptin resistance and overeating. In addition to HFD consumption, lipid accumulation is also observed in the ependymas of aging and Alzheimer’s disease (AD) mouse brains (Hamilton et al., 2015; Shimabukuro et al., 2016). Further research is needed to test how lipid droplets affect brain health.

In the ARC, leptin receptors are expressed in both AgRP and POMC neurons, and leptin depolarizes POMC neurons, stimulates α-melanocyte-stimulating hormone (α-MSH) secretion, simultaneously inhibits the activity of AgRP neurons and reduces the release of neuropeptide Y (NPY), AgRP, and γ-aminobutyric acid (GABA) (Waterson and Horvath, 2015). Insulin also promotes POMC neuron secretion but inhibits NPY/AgRP neurons (Dodd and Tiganis, 2017). In our data, we discovered that HFD feeding markedly decreased the amplitude of mEPSC in ARC neurons (Figures 3A–E) but had no effect on mIPSC (Figures 3F–J). Furthermore, HFD reduced the excitatory and inhibitory balance in the ARC (Figure 5H). These results indicate that HFD induced a decrease in excitatory input to the ARC but no significant change in inhibitory input, thus remodeling the E/I balance toward a more inhibitory direction. This may in part be caused by the lipid droplet disturbance of hormone penetration transmission, leading to damage to satiety signal delivery to POMC neurons and eventually promoting hyperphagia.

The VMH evokes sympathetic nervous system outflow and governs feeding behavior and blood glucose homeostasis (Tong et al., 2007). The VMH also expresses insulin and leptin receptors, but few studies explain their influence on the VMH. In the current research, HFD feeding reduced the amplitude of mEPSC but enhanced the frequency of that in the VMH (Figures 3K–O). We believe this was coordinated by multiple factors, including the direct and indirect effects of nutritional and hormonal abnormalities associated with HFD. Decreased postsynaptic responses corresponding to mEPSC amplitude changes may be in part due to lipid droplet accumulation-induced impairments of leptin and insulin penetration through the brain-CSF barrier. Furthermore, a previous study proposed that obesity enhances orexin fibers innervating targets in the VMH, and the increased orexin signaling overactivation of neurons in the VMH likely corresponds to the mEPSC frequency increase, which may lead to the obesity-related dysregulation of autonomic sympathetic nervous outflow (Cristino et al., 2013). The role of the VMH in obesity development needs further study.

Previous studies have shown that HFD-induced changes in spontaneous synaptic transmission are time-dependent in the LH. For example, orexin neurons display excitatory changes early in the HFD feeding period (1–4 weeks of HFD feeding) but normalize as the diet becomes chronic (11 weeks of HFD feeding) (Linehan et al., 2020). These data imply that short-term HFD exposure will likely lead to increased release of orexin peptide, which may activate downstream targets of the reward system, such as the VTA and NAc, then influence the reward value of palatable diets and reinforce further consumption by promoting motivation and food-seeking behavior (Yamanaka et al., 2003; Liu et al., 2020). However, when feeding becomes chronic, HFD-induced effects on orexin neurons reversed to normal levels. This may be due to damage to the reward system caused by dietary fat ingestion and diminished energy expenditure (Hryhorczuk et al., 2016). HFD was also found to affect the activity of other brain regions depending on feeding time. Such short-term HFD feeding does not inhibit leptin signals to AgRP neurons, while long-term HFD feeding abolishes the effects of leptin on AgRP neurons (Baver et al., 2014; Jais et al., 2020). Therefore, brain impairment from palatable food is a dynamic process, and the present study focuses on the effects of chronic HFD feeding. Elucidating whether and how excessive calorie intake causes adaptive changes in different brain regions is of particular interest. Investigating changes in synaptic plasticity over the course of HFD feeding are crucial to choosing the appropriate timing for weight-loss neural interventions.

PVH neurons are necessary for satiety and the prevention of obesity. Most PVH neurons expressed a transcription factor, single minded-1 (SIM1). Lesion of these neurons causes hyperphagia and obesity (Xi et al., 2012). Our data show that chronic HFD induces increased inhibitory synaptic transmission in the PVH. These findings suggest that HFD suppresses the satiety regulation of PVH, which may further promote hyperphagic obesity. However, the mechanisms need further study.

The NAc contributes to the hedonic impact of feeding behaviors (Van Bloemendaal et al., 2015). Previous studies have found that chronic HFD feeding reduces the expression levels of various receptors in the NAc, including D1 and D2 dopamine receptors, mu opioid receptors and the cannabinoid CB1 receptors. These receptors all affect hedonic eating (Gendelis et al., 2021). Human studies have shown a negative correlation between body fat and the volumes of gray matter in the NAc (Dekkers et al., 2019). Nevertheless, the effect of a HFD on the NAc is controversial. In our data, chronic HFD feeding reduced both the excitatory and inhibitory inputs to the NAc (Figures 5A–J). Taken together, chronic HFD feeding may reduce the circuit connections in the NAc. We speculate that obesity decreases the synaptic counts and impairs the synaptic plasticity of the NAc, which blunts the reward function of feeding and likely promotes eating more palatable food as compensation.

## Conclusion

In summary, we identified that HFD impairs synaptic transmission in hypothalamic feeding-related brain regions, including the ARC, VMH, LH, and PVH, which may be partly related to the accumulation of lipid droplet in the ependyma of the 3V. Otherwise, HFD feeding also damages synaptic transmission in the NAc, perhaps further attenuating food reward responses. Here, we show that HFD-induced alteration of synaptic transmission in feeding-related brain regions provides a potential explanation for overeating and hedonic eating in obese individuals and that targeting these regions is promising as a therapeutic strategy for hyperphagic obesity.

## Data availability statement

The raw data supporting the conclusions of this article will be made available by the authors, without undue reservation.

## Ethics statement

All experiments were performed in accordance with the guidelines of the Institute of Neuroscience, Chinese Academy of Sciences and University. All procedures involving animals were approved by the Hubei Provincial Animal Care and Use Committee and were in accordance with the experimental guidelines of the Animal Experimentation Ethics Committee of Huazhong University of Science and Technology, China.

## Author contributions

HL designed and supervised the study. XW performed the experiments. HL and XW wrote the manuscript. Both authors contributed to the article and approved the submitted version.

## Footnotes

1
https://www.idtracker.es

2
https://imagej.nih.gov/ij/
