# Supplementary figures and images for: Chronic high-fat diet induces overeating and impairs synaptic transmission in feeding-related brain regions
Source: Front Mol Neurosci. 2022 Sep 26;15:1019446. doi: 10.3389/fnmol.2022.1019446 (PMC9549200; doi:10.3389/fnmol.2022.1019446)

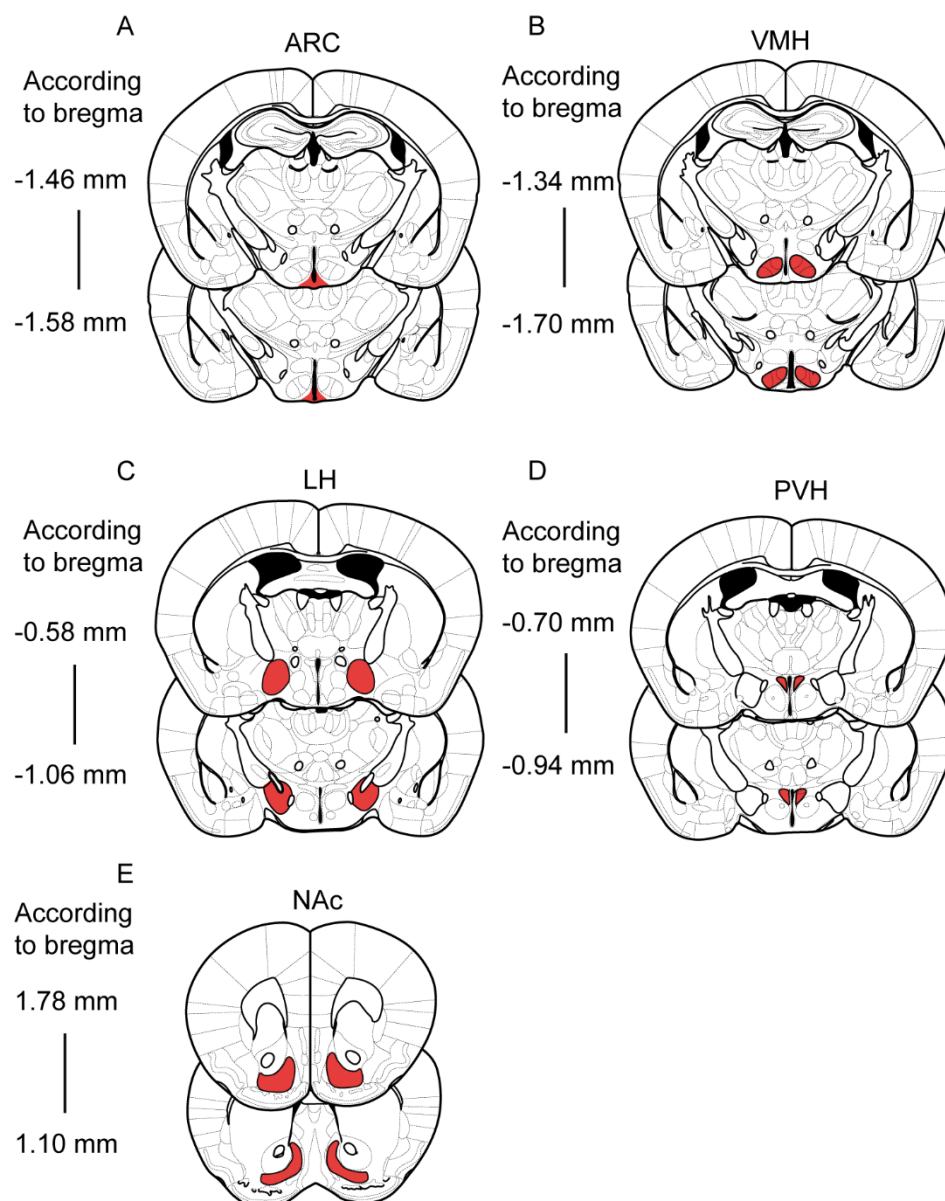

**FIGURE S1.** Recording sites of the ARC (A), VMH (B), LH (C), PVH (D), and NAc (E).

Supplement: Supplementary file 1 [file Presentation_1.pdf]
